# HTLV-1 clonality in adult T-cell leukaemia and non-malignant HTLV-1 infection

**DOI:** 10.1016/j.semcancer.2013.11.003

**Published:** 2014-06

**Authors:** Charles R.M. Bangham, Lucy B. Cook, Anat Melamed

**Affiliations:** Section of Immunology, Imperial College, London W2 1PG, UK

**Keywords:** HTLV-1, Retrovirus, Leukaemia, Lymphoma, Persistent infection, Clonality, Integration, High-throughput sequencing

## Abstract

Human T lymphotropic virus type 1 (HTLV-1) causes a range of chronic inflammatory diseases and an aggressive malignancy of T lymphocytes known as adult T-cell leukaemia/lymphoma (ATLL). A cardinal feature of HTLV-1 infection is the presence of expanded clones of HTLV-1-infected T cells, which may persist for decades. A high viral burden (proviral load) is associated with both the inflammatory and malignant diseases caused by HTLV-1, and it has been believed that the oligoclonal expansion of infected cells predisposes to these diseases. However, it is not understood what regulates the clonality of HTLV-1 in vivo, that is, the number and abundance of HTLV-1-infected T cell clones. We review recent advances in the understanding of HTLV-1 infection and disease that have come from high-throughput quantification and analysis of HTLV-1 clonality in natural infection.

## 1 Introduction

Human T lymphotropic virus type 1 (HTLV-1) is a retrovirus that is widespread in the tropics and sub-tropics. The total number of people infected is at least 5 to 10 million, but the true number is very uncertain, owing to incomplete epidemiological studies in the endemic regions [1].

HTLV-1 and its congeners HTLV-2, 3 and 4 [2] are retroviruses that belong to the *Deltaretrovirus* genus of the subfamily *Orthoretrovirinae*, while the other pathogenic human retroviruses HIV-1 and 2 are classified in the subfamily *Lentivirinae*. Unlike HIV-1 and 2, HTLV-1 does not cause disease in the majority (over 90%) of infected individuals. Between 1 and 4% of HTLV-1-infected people develop a chronic inflammatory disease, of which the commonest is HTLV-1-associated myelopathy/tropical spastic paraparesis (HAM/TSP), which causes progressive paralysis of the legs [3]. Some 5% of HTLV-1-infected individuals develop adult T cell leukaemia/lymphoma (ATLL), a T cell malignancy with a characteristically poor prognosis [4].

The history [5], [6] and epidemiology [1], [7], [8] of HTLV-1 have been ably reviewed elsewhere. The purpose of the present review is to consider two questions. First, what regulates the clonality of HTLV-1 in vivo, that is, the selective outgrowth of certain clones of T cell infected with HTLV-1? Second, what is the role of this oligoclonal proliferation in the pathogenesis of the inflammatory and malignant diseases associated with HTLV-1? A clone of HTLV-1-infected T cells is identified as a population of cells that carry the HTLV-1 provirus integrated at the same site in the host genome.

## 2 Adult T cell leukaemia/lymphoma (ATLL)

ATLL is a malignancy of mature, post-thymic T lymphocytes [9]. ATLL cells have a characteristic morphology, with a large, multi-lobed nucleus, giving rise to the epithet “flower cell”. In the great majority of cases, the phenotype of the malignant cell is CD4^+^ CD8^−^; about 4% of cases are CD4^−^ CD8^+^, and a similar proportion CD4^+^ CD8^+^ or CD4^−^ CD8^−^ [10]. The cells usually express the markers CD2 and CD5; CD3 and TCRß are frequently downregulated or undetectable at the cell surface. The cells also express several molecules that are characteristic of regulatory T cells, including the cell surface molecules CD25, CCR4, GITR and the transcription factor FoxP3. However, these molecules are also expressed by activated T cells, and it appears that ATLL is not per se a malignancy of regulatory T cells [11].

ATLL was classified into 4 clinical subtypes by Shimoyama et al. [12], according to the lymphocyte count, serum calcium concentration, lactate dehydrogenase level, solid organ involvement and the severity of systemic symptoms. The most common acute form (about 65% of cases) can present as a medical emergency, with bulky lymphadenopathy, a florid and rapidly increasing leukocytosis, hypercalcaemia, frequently with destructive bone lesions, dehydration, and severe systemic symptoms. In the chronic form, the lymphocytosis can also be very marked (over 50 × 10^9^ cells L^−1^), but the cell count rises more slowly, and the patient can remain stable with minor or absent symptoms for months or even years. A proportion of cases (∼20%) present as a lymphoma, with a normal circulating lymphocyte count. This diagnostic classification remains useful for purposes of standardizing clinical trials, comparing disease and treatment outcomes between centres, choosing appropriate therapy and for assessing the prognosis. However, the classification does not reflect the continuum of presentation in the clinic. For example, a purely cutaneous form of ATL lymphoma is recognized, which occurs without leukaemic or nodal disease, and which carries a substantially better prognosis than nodal lymphomas.

## 3 Treatment

ATLL carries a poor prognosis because of intrinsic chemotherapy resistance and severe immunosuppression. Despite advances in medical management and supportive care, chemotherapy trials report a median survival of the aggressive subtypes between 7 and 13 months [13], [14], [15]. Clinical trials of combination chemotherapy in acute ATLL have achieved improved response rates but have not prolonged survival. Patients with indolent forms of ATLL have a better prognosis (median overall survival 4.1 years [16]) but the long-term survival remains poor when managed with either watchful waiting or conventional chemotherapy. A recent meta-analysis of non-Japanese patients treated with zidovudine and IFNα revealed this to be a highly effective treatment for leukaemic subtypes of ATLL [17]. Lymphoma subtypes may still benefit from chemotherapy, with either concurrent or sequential zidovudine + IFNα treatment to prevent relapse [18]. The risk of relapse with all ATLL subtypes remains high and the role of consolidation treatment with immunomodulatory therapies such as zidovudine + IFNα, arsenic trioxide or with monoclonal antibodies such as basiliximab or mogamulizumab is yet to be established. Allogeneic bone marrow transplantation remains the only curative option but is only possible in those individuals who achieve a complete response to treatment, have an HLA-matched donor and are physically fit for the procedure.

## 4 HTLV-1 molecular virology

**Fig. 1:**
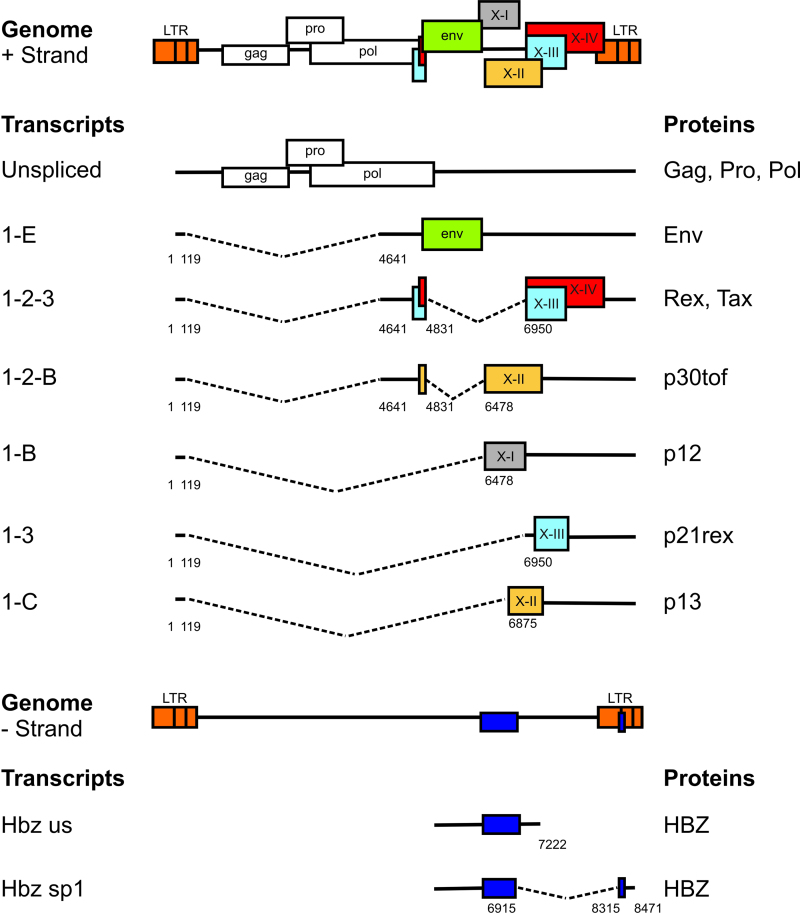
Structure and coding potential of plus- and minus-strand HTLV-1 mRNAs. ^©^The American Society of Hematology.

Reproduced with permission from Figure 1a in Rende et al. [95].

The genome of HTLV-1 and the major transcripts are shown in Fig. 1. In addition to the *gag*, *pol* and *env* gene products found in other exogenous replication-competent retroviruses, HTLV-1 encodes at least 7 regulatory gene products which control the proviral transcription, mRNA splicing and transport, and the expression of certain host genes. The functions of these regulatory genes of HTLV-1 have been reviewed elsewhere [19], [20]. Among these genes, two, *tax* and *HBZ*, appear to play a particularly important role in regulating the expression of viral and host genes and the activation and proliferation of the host cell [20], [21]. The transcriptional transactivator Tax recruits host cell transcription factors, notably CBP/p300, and activates transcription of the virus itself, from the promoter/enhancer in the 5′ long-terminal repeat (LTR) (Fig. 1), creating a strong positive feedback loop. In addition, Tax activates the NF-κB and AKT pathways, thereby upregulating many host genes [22]. This widespread gene activation results in activation and proliferation of the host cell [20], [23] and transmission of HTLV-1 to other host cells via the virological synapse [24], [25].

HTLV-1 Tax protein has a remarkable range of actions on the host cell, promoting DNA replication and cell-cycle progression, structural damage to the host cell DNA, inhibition of DNA repair and cell-cycle and DNA damage checkpoints, and centrosome over-duplication. Understandably, Tax has therefore been believed to be necessary and sufficient to cause ATLL. Tax is indeed sufficient to immortalize rat fibroblasts in culture, and Tax-transgenic mice develop a variety of tumours [26], [27], [28]. However, mouse cells appear to be transformed more readily than human cells [29], and attempts to transform human cells in vitro with Tax have failed.

A second paradox concerning the putative oncogenic role of Tax is the fact that some 60% of ATLL clones do not express Tax, although the transformed cell typically retains the phenotype (CD25^+^ FoxP3^+^ GITR^+^, etc.) of the Tax-expressing cell. The loss of Tax results from one of 3 mechanisms: deletion or methylation of the 5′ LTR, or mutation of the provirus [20], [21]. It is thought that the pressure to lose Tax expression is exerted by the strong host cytotoxic T lymphocyte (CTL) response to the Tax protein [30].

In 2002 a new gene was discovered in HTLV-1 [31]. The HTLV-1 bZIP factor, HBZ, is expressed from the negative strand of the provirus (Fig. 1), driven by the transcription factor Sp1 from a promoter in the 3′ LTR. In contrast with Tax, HBZ appears to be expressed at a constant (albeit low) level in most if not all HTLV-1-infected cells, both non-transformed and malignantly transformed [32].

HBZ has important actions at both the protein and mRNA levels [20]. HBZ protein can reduce Tax-mediated viral transcription by heterodimerizing with Jun and CREB2 [33]. HBZ also selectively inhibits activation of the classical NF-κB pathway [34]; since Tax activates both the classical and alternative pathways of NF-κB, it is possible that chronic activation of the alternative NF-κB pathway by persistent HBZ expression plays a part in the proliferation of HTLV-1-infected cells in vivo [20]. This interpretation is favoured by the observation that an efficient CD8^+^ T-cell response to HBZ is associated with a lower proviral load and a lower risk of the inflammatory disease HAM/TSP [35], [36]. HBZ mRNA, rather than the protein, promotes expression of the transcription factor E2F1, supports proliferation of ATLL cells in vitro [32], increases the proviral load of HTLV-1 in the rabbit [37], and increases the activity of the telomerase hTERT [38].

## 5 Cellular tropism and propagation of HTLV-1

HTLV-1 can infect virtually all nucleated mammalian cells in vitro [39], but in vivo it is almost confined to T lymphocytes and dendritic cells (DCs) [25], [40]. Typically about 95% of the proviral load – the proportion of circulating mononuclear leukocytes infected – is carried in CD4^+^ (helper/regulatory) T cells, and 5% in CD8^+^ T cells [40] (AM, unpublished data). DCs constitute a very small fraction of the load, but it is possible that they play a disproportionate role in propagating the virus within one host, particularly in the early stages of infection, because of their high mobility and their propensity to form intimate contacts with other cells [41], [42]. HTLV-1 releases almost no cell-free virus particles in vivo. Instead, when an infected cell makes contact with another cell, a synergistic interaction between extracellular and intracellular signals leads to cytoskeletal polarization in the infected cell and causes directed assembly and budding of the virus at the cell-to-cell contact, resulting in efficient transfer of the virus to the “target” cell [24]. This specialized, virus-induced cell-to-cell contact is known as a virological synapse [24]. Thus, the virus exploits the mobility of the host cell instead of releasing mobile extracellular particles. As a result, cell-free blood products from HTLV-1-infected people are not infectious; HTLV-1 is transmitted between individuals by transfer of infected leukocytes in breast milk, semen or blood [7].

### 5.1 What determines the equilibrium proviral load in an individual with HTLV-1 infection?

Early studies found no systematic association between HTLV-1 genotype and disease manifestation [43], [44], [45]. In 2000, Furukawa and his colleagues reported [46] a higher prevalence of HAM/TSP among people in southern Japan infected with the cosmopolitan subtype A of HTLV-1. However, the strongest correlate of disease risk [47], [48] and progression [49] is the proviral load, i.e. the fraction of peripheral blood mononuclear cells (PBMCs) that carry the HTLV-1 provirus. The proviral load can reach remarkably high levels, frequently over 10% of PBMCs, i.e. over 20% of CD4^+^ T cells, the main host cell. Within each host the proviral load remains stable over time [49], but varies by more than 1000-fold among hosts. The question arises: what determines the proviral load set point in a given host?

Like other exogenous, replication-competent retroviruses, HTLV-1 can propagate both by proliferation of the provirus-carrying cell (“mitotic spread”) and by de novo virion production (“infectious spread”) [50]. As described above, cell-free virions are undetectable in vivo. In the chronic phase of infection HTLV-1 persists chiefly by mitotic spread, i.e. by proliferation of T cells that carry an integrated provirus of HTLV-1. The evidence for this comes from two main observations. First, the peripheral blood contains expanded T cell clones that carry HTLV-1 in the same genomic integration site [51], [52], [53], [54]: such clones can persist for years in the host [53], [54], [55]. Second, HTLV-1 varies little in sequence both within and between hosts [43], [44], [45], in sharp contrast with HIV-1, and the rate of evolution of HTLV-1 is low compared with other retroviruses [56], [57]: these observations suggest that the error-prone enzyme reverse transcriptase [58] contributes relatively little to the replication of HTLV-1 during chronic infection [59], [60].

Oligoclonal expansion of HTLV-1-infected lymphocytes in vivo is frequently easier to detect in patients with HAM/TSP than in asymptomatic HTLV-1 carriers (ACs) [54], and monoclonal expansion is a defining feature of ATLL [61]. It has therefore been presumed that oligoclonal proliferation plays a causative role in both the inflammatory and malignant diseases caused by HTLV-1. However, it has not been clear whether the apparently greater oligoclonality observed in HAM/TSP was an artefact of the relatively insensitive methods used to detect and quantify the clones: both linker-mediated and inverse PCR and genomic Southern blotting can reproducibly identify only relatively abundant clones.

## 6 A strong cytotoxic T-cell response limits HTLV-1 proviral load and the risk of HAM/TSP

Since HTLV-1 varies little in sequence, and the same viral sequence can occur in asymptomatic HTLV-1 carriers (ACs) and patients with HAM/TSP or ATLL, the observed variation in the outcome of infection among individuals must be chiefly due to variation in the host. There is strong evidence that the principal determinant of an individual's proviral load and risk of HAM/TSP is the HLA Class 1-associated CD8^+^ cytotoxic T lymphocyte (CTL) response to HTLV-1. This evidence comes from experiments in host genetics [62], [63], [64], viral genetics [65], lymphocyte gene expression [66], assays of lymphocyte function [67], [68], and mathematical analysis [23], [59], [69]. Consistent with this notion, the protective host gene HLA-A*02 was found to give less protection against HAM/TSP in individuals infected with the Cosmopolitan subtype A of HTLV-1 which, as noted above, was associated with a higher prevalence of HAM/TSP in Japan [46].

The HTLV-1 transactivator protein, Tax, is highly immunodominant in the CTL response to HTLV-1 [70], [71]. However, we recently found that the proviral load and the risk of HAM/TSP are determined by the CTL response to a subdominant antigen, HBZ, and not by the response to Tax [35], [36]. The picture is emerging that the regulation of *tax* and *HBZ* expression from the provirus plays a central role in the persistence and pathogenesis of HTLV-1 infection [20].

To summarize: since both *tax* and *HBZ* gene products promote proliferation of the infected cell, both have been suggested as necessary and sufficient causes of both the oligoclonal T cell proliferation seen in HTLV-1 infection and the pathogenesis of inflammatory and malignant diseases associated with HTLV-1. The potential pathogenic role of these viral gene products must be understood in the context of their normal physiological function in the life history of HTLV-1, since the primary function of these viral genes is not to cause disease in the host but rather to promote survival and propagation of the virus. The central question therefore becomes this: what regulates the expression of the *tax* and *HBZ* genes in vivo, and so controls the number, abundance and pathogenicity of HTLV-1-infected T cell clones in vivo?

To answer this question, we must consider what differs between two clones of T cells naturally infected with HTLV-1. There are three principal attributes that distinguish one infected T cell clone from another: antigen (TCR) specificity, epigenetic modifications, and the genomic site of integration of the HTLV-1 provirus. In addition, as a consequence of the epigenetic modifications, there may be differences among clones in the expression of certain cell surface markers. We have hypothesized that the chief factor that regulates the expression of the HTLV-1 provirus is the integration site of the provirus in the host genome. To test this hypothesis, we recently developed [72] a sensitive, high-throughput technique for the mapping and – crucially – quantification of HTLV-1-infected T cell clones in fresh uncultured peripheral blood mononuclear cells (PBMCs). We have used this protocol to address the following questions:

• How many proviruses are present in each cell?
• How many distinct HTLV-1^+^ clones are present in a single host?
• What regulates the abundance of a given clone in vivo?
• What regulates expression of the provirus by a given clone?
• Does oligoclonal proliferation contribute to the pathogenesis of HTLV-1-induced diseases?

## 7 High-throughput mapping and quantification of retroviral integration sites

The high-throughput integration site protocol [72] consists of PCR amplification of genomic DNA fragments to which a partially double-stranded DNA linker has been ligated. The protocol differs in a critical respect from preceding high-throughput retroviral mapping techniques. Instead of using restriction enzymes to digest the genomic DNA before linker ligation, the DNA is fragmented by sonication. The resulting quasi-random distribution of DNA fragment lengths confers two crucial advantages. First, it abrogates the biased detection – due to preferential amplification of short fragments – of proviruses integrated close to a given restriction enzyme site. Second, since the DNA shear sites are virtually random, each sister cell of a given HTLV-1-infected T-cell clone can be identified by the unique length of the amplicon. In this way, the sister cells belonging to each clone can be enumerated and the clonal abundance can be accurately estimated [73].

### 7.1 How many proviruses are present in one cell?

We isolated 28 clones of naturally infected T cells by limiting dilution from the peripheral blood of patients with non-malignant cases of HTLV-1 infection [74]. The clones were expanded in vitro in the presence of the integrase inhibitor raltegravir, to minimize secondary spread of the virus. We then used the high-throughput protocol to quantify the number of HTLV-1 provirus integration sites present in each clone. The results showed that every clone examined carried a single integrated provirus. These results do not exclude the possibility that some clones carry more than one integrated provirus in vivo, but suggest that such clones are in the minority in non-transformed cells. However, the incidence of multiple integration sites may be higher in ATLL clones than in non-transformed clones [75], [76], [77].

Josefsson et al. [78] recently reported evidence, using a different approach, that single integrated proviruses also predominate in HIV-1 infection. The finding that the majority of naturally-infected clones carry a single provirus in both HIV-1 and HTLV-1 infection is surprising. Since both HIV-1 and HTLV-1 are transmitted more efficiently by cell-to-cell contact than by free virions, and indeed this appears to be virtually the exclusive route in HTLV-1 infection, one might expect that several virions would enter the newly-infected cell and result in several proviral integrations, each in a different genomic location. These observations therefore suggest that specific mechanisms exist to limit the number of proviruses that integrate in one cell. This phenomenon of superinfection resistance in retroviruses is well described [79], but the molecular mechanisms are not fully explained.

### 7.2 How many distinct HTLV-1-infected clones are present in one host?

In ATLL, a single HTLV-1-infected clone typically dominates the viral population. In non-malignant cases of HTLV-1 infection, the disproportionate expansion of certain infected T cell clones was first detected by Southern blotting of genomic DNA and by linker-mediated PCR (LM-PCR) [53]. These early experiments led to the estimate that a typical host with HTLV-1, without ATLL, carries about 100 clones of HTLV-1-positive lymphocytes in the circulation [52]. However, these techniques are at best semi-quantitative and, more importantly, have a limited dynamic range. That is, a single clone must be present at high frequency to be reproducibly detected by these methods, but a highly abundant clone is difficult to distinguish from a merely detectable clone. As a result, neither the number nor the absolute or relative abundance of clones could be reliably estimated by such techniques. The new high-throughput protocol has changed the understanding of HTLV-1 clonality in vivo. Typically, thousands of distinct integration sites are detected in 10 μg of genomic DNA from peripheral blood mononuclear cells [72], [80]. We are now developing a curve-fitting technique to extrapolate the observed frequency distribution of clones in a given individual, in order to estimate the total number of clones present in the circulation of that individual. The results (Laydon et al., submitted for publication) indicate that the median number of distinct HTLV-1-positive clones in the circulation lies between 20,000 and 50,000. The lymphocytes in the circulation represent only 2% of the number in the whole body, but the relationship between the clone frequency distribution in the blood and in solid lymphoid tissues remains unknown. If we assume that the frequency distribution in the blood represents the frequency distribution in the solid lymphoid tissues, the estimated number of HTLV-1^+^ clones rises to >60,000.

How long does each HTLV-1^+^ T cell clone live in vivo? It was already clear from the pioneering work of Wattel and colleagues [52], [53] that individual clones could persist for many years. Data from the high-throughput protocol corroborate this finding [72]. Further work is now in progress, to estimate the longevity of the previously undetectable, low-abundance clones, in order to answer the question: what is the contribution of de novo infection to the maintenance of the proviral load during persistent infection? That is, what is the ratio of mitotic spread to infectious spread [50]? The answer to this question will determine the potential to limit viral propagation in the host by using either anti-mitotic drugs, to inhibit proliferation of HTLV-1-infected cells, or anti-retroviral drugs, to inhibit the production of new infected T cell clones.

### 7.3 What determines the site of proviral integration?

Retroviral integration into the host genome is not random [81], but is biased at 3 distinct levels. First, the chromatin structure is critical: integration is biased towards euchromatin [72], [82], whose open conformation allows the retroviral preintegration complex access to the DNA. Second, at the primary DNA sequence level, integration is biased towards a short nucleotide motif [83], [84], whose palindromic nature is consistent with the two-fold symmetry of the retroviral integrase [85], [86]; the length and sequence of the motif are specific to each retrovirus. Third, retroviral integration is not equally frequent in all euchromatic sites that possess this palindromic motif, but is biased by an interaction between the preintegration complex and specific host factors. The best characterized of these host factors is LEDGF [87], which strongly biases the integration of HIV-1 into genes and away from intergenic regions. Certain other host factors also influence integration site selection in HIV-1 infection, including HRP-2 [88], and Transportin-3 and RanBP2, which appear to link integration to transport of the pre-integration complex into the nucleus [89]. The transcription factor YY1 similarly plays a role in guiding the integration of murine leukaemia virus [90], but in most retroviral infections, including HTLV-1, the putative integrase-interacting host factors have not been identified.

To identify the factors associated with targeting of HTLV-1 integration, we investigated the characteristics of the host genome flanking the integrated provirus, using the high-throughput quantitative protocol [72], [80]. To distinguish between the integration sites favoured in initial targeting and the sites that survive selection during persistent infection in vivo, we studied the integration sites after short-term in vitro infection of human lymphocytes and in PBMCs from people with different manifestations of HTLV-1 infection. The results demonstrated the expected predominance of integration sites in transcriptionally active euchromatin, as indicated by the frequency of epigenetic marks associated with transcriptional activity [72]. In addition, there was a remarkably strong bias towards integration within 100 base-pairs of certain transcription factor binding sites, especially binding sites for the tumour suppressor P53 and the transcriptional regulator of interferons, STAT1: in each case, an integrated HTLV-1 provirus was between 100-fold and 350-fold more likely to lie within 100 base-pairs of the respective binding site than expected by chance [80]. Integration targeting of HTLV-1 was also significantly (but less strongly) associated with several other sites that bind specific transcription factors or chromatin-modifying factors, such as SWI/SNF. The mechanism of specific targeting of these sites is unexplained, and requires identification of the host factors that interact with HTLV-1 integrase.

### 7.4 What determines spontaneous proviral expression?

The selective oligoclonal expansion of certain HTLV-1-infected T cell clones is a cardinal feature of both non-malignant HTLV-1 infection and, by definition, the malignant disease ATLL. We postulated that the proviral integration site determines the pattern – i.e. the frequency and intensity – of spontaneous proviral expression, which in turn determines the selective expansion of particular HTLV-1^+^ clones.

Fresh unstimulated PBMCs taken from an HTLV-1-infected person usually do not express detectable levels of HTLV-1 antigens, but strong Tax protein expression becomes detectable after about 6 hours’ incubation in vitro [91]. We previously showed that these spontaneously Tax-expressing cells belong to clones that proliferate more frequently than non-Tax-expressing cells in vivo [23]. To identify the characteristics of the proviral integration site associated with spontaneous Tax expression, we isolated the Tax-expressing cells by flow cytometry and compared the integration sites between the Tax-positive and Tax-negative cells [80].

The results [80] showed that proviral integration within 100 nucleotides of genomic binding sites for certain transcription factors or chromatin-modifying factors was strongly associated with spontaneous Tax expression; some of these factors (e.g. STAT1) were also associated with integration targeting (see above). However, there was a critical difference between the pattern of association with targeting and that associated with Tax expression: whereas the binding sites associated with integration targeting were distributed symmetrically about the integration site, the binding sites associated with the spontaneous expression were frequently asymmetrical. For example, a STAT1 binding site lying 10 or 100 base-pairs upstream of the HTLV-1 provirus was associated with spontaneous Tax expression, but a STAT1 site lying a similar distance downstream had no effect. The strongest and most unexpected effect was that of BRG1, an ATPase that powers the chromatin remodelling complex SWI/SNF. Whereas the presence of a BRG1 site (identified by ChIP) 10–100 base-pairs upstream was associated with silencing of Tax expression, a BRG1 site 10–100 base-pairs downstream of the provirus was associated with spontaneous Tax expression. The asymmetry of these effects strongly implies that these DNA binding sites are not associated with proviral expression simply by virtue of lying in open-conformation chromatin. Rather, the asymmetry implies a mechanistic interaction between transcription of the provirus and transcription of the flanking host genome. This conclusion was reinforced by the observation [80] that the transcriptional orientation of the provirus relative to the nearest host gene was also associated with the frequency of spontaneous expression of the provirus. We expected that a provirus lying downstream of the host transcriptional start site and in the same transcriptional sense would be more likely to express Tax than a provirus lying in the opposite transcriptional orientation. But the results showed exactly the opposite effect: a same-sense host transcriptional start site upstream appeared to suppress Tax expression, whereas a same-sense transcriptional start site downstream of the provirus was associated with spontaneous Tax expression.

The observation that Tax expression is suppressed by the presence upstream of either chromatin remodelling factors or an active host transcriptional start site strongly suggests that the dominant interaction between the flanking host genome and the provirus is transcriptional interference: that is, the inhibition of transcription of the provirus from the 5′ LTR by the presence of an active nearby host promoter upstream of the provirus. It is probable that transcriptional interference contributes to silencing of other integrated proviruses, and it may therefore help to maintain the reservoir of latent HIV-1 [92]. The mechanisms of transcriptional interference are not fully understood; one possible mechanism is occlusion of the downstream promoter by an active transcription complex, a phenomenon called promoter occlusion.

### 7.5 What determines HTLV-1 clonal abundance in vivo?

It has been widely believed that oligoclonal expansion of HTLV-1-infected T cells is not only responsible for persistence of the infection in vivo but also maintains the high proviral load and predisposes to both inflammatory and malignant diseases associated with HTLV-1. A strong advantage of the recently developed high-throughput proviral sequencing protocol is the ability to quantify accurately the abundance of each HTLV-1-infected T cell clone. This in turn makes it possible to identify the factors associated with the selective expansion of certain clones in vivo. We found that the chief determinants of clonal abundance were the transcriptional orientation of the provirus and its position (upstream or downstream), relative to the nearest host transcriptional start site. Proviruses integrated within a host gene were significantly more frequent in clones of high abundance in vivo than in those with low abundance, but only when integrated in the same transcriptional sense as the host gene.

**Fig. 2:**
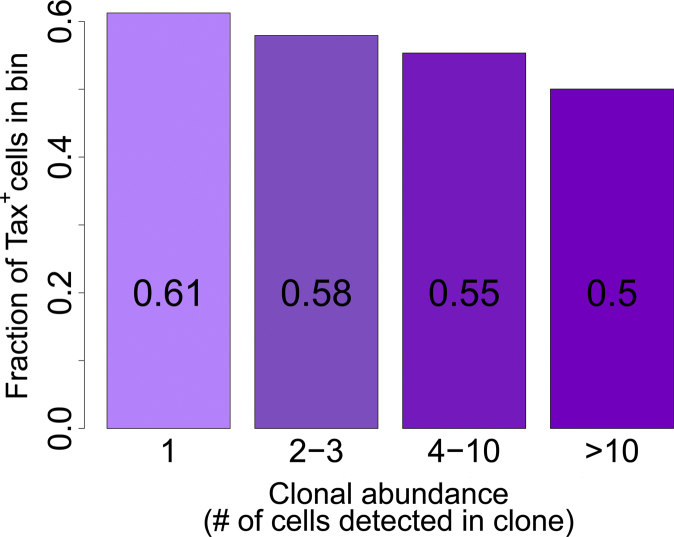
The fraction of cells in each HTLV-1-infected T-cell clone that spontaneously expresses the Tax protein is negatively correlated with the abundance of the clone in vivo (*P* < 10^−16^, chi-squared test for trend).

Reproduced from Figure 6 in Melamed et al. [80].

Because of the known mitogenic properties of Tax, we postulated that Tax-expressing clones would reach a higher mean abundance than non-expressing clones in the circulation. But again the results confounded expectation: the frequency of Tax expression was significantly greater in low-abundance clones (Fig. 2) [80]. Although it was unexpected, this result is consistent with the observations noted above that orientation of the provirus in the same transcriptional sense as the flanking host gene is associated with silencing of Tax expression [80] and with high clone abundance [72], [80].

### 7.6 Is oligoclonal expansion associated with inflammatory diseases such as HAM/TSP?

Since the proviral load is higher in HAM/TSP patients than in asymptomatic HTLV-1 carriers, and oligoclonal proliferation is frequently detected more easily in samples from patients with HAM/TSP [54], it was natural to infer that oligoclonal proliferation was stronger in HAM/TSP and therefore that it might contribute to the pathogenesis of the inflammatory disease. However, this inference could not be formally tested in the absence of an objective measure of oligoclonality. What is required is a measure of the non-uniformity or entropy of the clone frequency distribution.

**Fig. 3:**
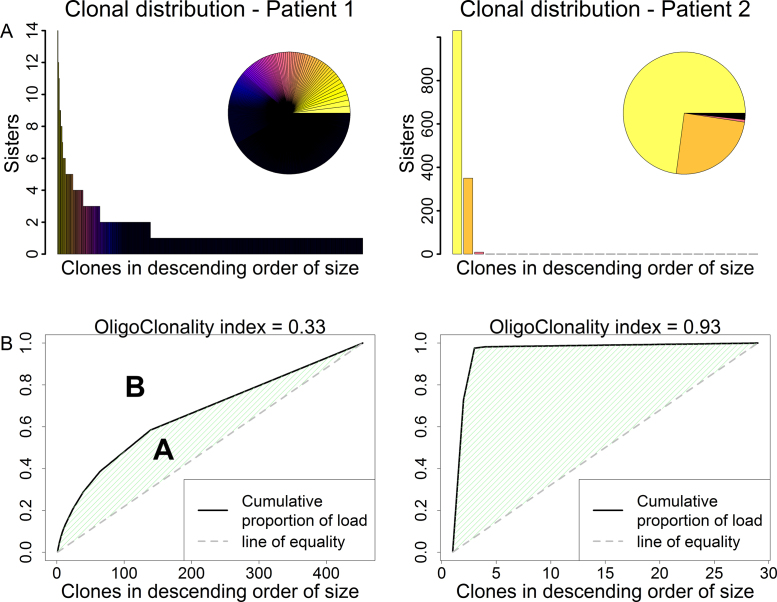
Clone frequency distribution of HTLV-1-infected T cells in a representative subject (Patient 1) with non-malignant HTLV-1 infection and a representative case (Patient 2) of ATLL. Upper panels depict the number of cells detected in each clone in a sample of genomic DNA from peripheral blood mononuclear cells, in descending order of clone abundance. In the inset pie charts, the size of each sector depicts the relative abundance of the respective clone; the solid black sector denotes a large number of low-abundance clones. Lower panels show the cumulative fraction of the proviral load constituted by the infected T-cell clones. The oligoclonality index, OCI, is defined [72] as OCI = *A*/(*A* + *B*).

A widely used entropic index, the Shannon index, is of very limited usefulness here because this index is correlated with the sample size, which can be very large in high-throughput sequencing. We therefore defined [72] the oligoclonality index (OCI), an application of the Gini index (Fig. 3). An OCI of 1 indicates perfect monoclonality, whereas an index of 0 indicates that each clone has the same frequency. This index allows a rigorous quantitative comparison of the degree of oligoclonality between disease states. We found that, contrary to expectation, there is no significant difference in oligoclonality (as measured by OCI) between patients with HAM/TSP and asymptomatic carriers [72]; The OCI in patients with malignant disease, ATLL, is significantly higher, as expected. Further, the degree of oligoclonality (OCI) does not correlate with the proviral load in patients with non-malignant infection [72]. Rather, the proviral load correlated with the total number of distinct clones, and this number is significantly greater in patients with HAM/TSP than in asymptomatic carriers.

These observations lead to the general conclusion that oligoclonal proliferation is not a major contributor to the pathogenesis of the associated diseases: clonal expansion is a feature of established ATLL but the expansion per se is not responsible for the malignant transformation. Rather, the proviral load and the risk of inflammatory or malignant disease are determined by the large number of low-abundance clones: these are the clones that frequently express Tax [80] and turn over rapidly in vivo [23]. The principal factor that limits the abundance and the number of these cells in vivo is the genetically-determined efficiency or ‘quality’ of the host CTL response to the virus [30], particularly to the HBZ protein [36].

### 7.7 HTLV-1 clonality in ATLL

**Fig. 4:**
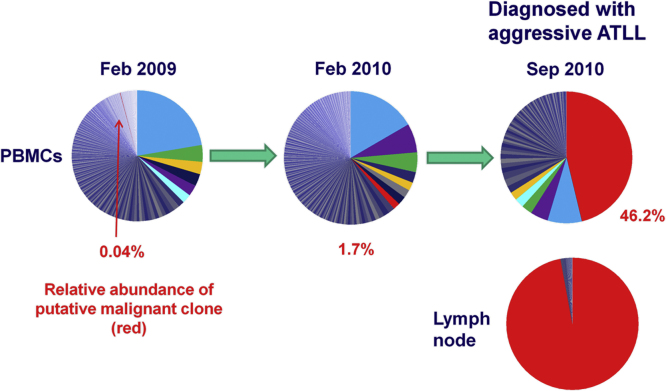
Rapid emergence of ATLL. In February 2009, one clone (shaded blue) dominated the infected cell population present in the peripheral blood of an infected person. However, the clone that underwent malignant transformation to cause ATLL 18 months later was a minor clone, occupying only 0.04% of the proviral load at this date. Such cases suggest that clonal expansion per se of HTLV-1^+^ cells does not predispose to malignant transformation.

The understanding of clonality in ATLL is less advanced than in non-malignant HTLV-1 infection, and further work is required. It is widely assumed that ATLL is a monoclonal disease, and indeed in a typical case of acute ATLL a single clone usually dominates. However, there are indications that clonality in ATLL is not always simple. First, there are often many HTLV-1-infected T cell clones underlying the largest, putatively malignant clone [72] (LBC, unpublished data); not infrequently, more than one clone appears to be abnormally abundant and is presumed to be malignant. Second, the malignant clone does not necessarily develop from the largest pre-existing infected T cell clone, but can develop rapidly from a clone of previously very low abundance (Fig. 4). Third, there are well-described instances of “clonal succession”, in which a putatively malignant clone spontaneously regresses and another clone takes its place [77]. Subclonal diversification of cells from a single common ancestor is well described in solid tumours (reviewed by Vogelstein et al. [93]). In contradistinction, the evidence suggests that ATLL can be a polyclonal tumour, i.e. with more than one independently transformed cell of origin.

**Fig. 5:**
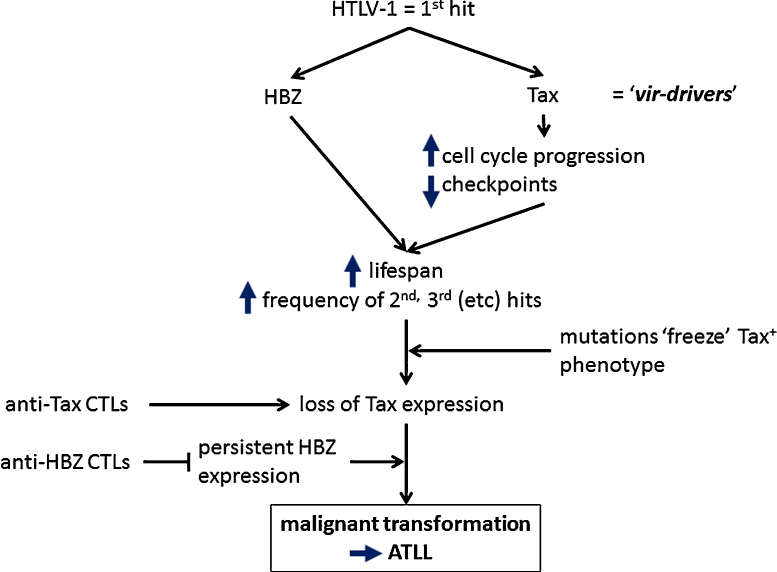
Model of the development of ATLL. HTLV-1 infection constitutes the first hit that predisposes each infected T-cell to malignant transformation. The probability that a given clone undergoes transformation depends principally on the balance of two opposing forces: negative selection by the host CTL response to HTLV-1 antigens, and persistent or repeated expression of two regulatory genes of HTLV-1, *tax* and *HBZ*, whose products prolong the lifespan of the T-cell clone and so increase the chance of accumulation of further genetic or epigenetic hits. These further hits may cause constitutive activation of the pathways initially activated by Tax, after which loss of Tax expression confers a survival advantage on the clone by escape from the strong anti-Tax CTL response. In contrast to the frequent silencing of Tax expression, HBZ expression appears to persist [32]; an effective CTL response to HBZ reduces the proviral load and the risk of HAM/TSP, and may reduce the risk of ATLL.

We postulate that HTLV-1 constitutes the first ‘hit’ of the 5–8 hits – usually an alteration in a driver gene – that are thought to cause malignant transformation [94]. Consequently, every HTLV-1-infected T cell lies on a spectrum of risk of undergoing transformation. Perhaps the simplest hypothesis is that the risk of malignant transformation of an HTLV-1-infected T cell depends chiefly on the longevity of that clone and, in particular, the total number of cell divisions the clone has undergone. The longevity of the clone in turn depends on the pattern of proviral expression, which in ideal circumstances maintains the cell in cycle while minimizing its exposure to host CTL surveillance. A simplified scheme of the proposed sequence of events in the pathogenesis of ATLL is shown in Fig. 5. The consequences of HTLV-1 gene products that promote malignant transformation, such as DNA damage, are presumably merely side-effects of mechanisms that favour clone survival in vivo.

The intense current effort in cancer genomics has led to the identification of a relatively small number of mutations that drive malignant transformation, as distinct from the adventitious and passively propagated “passenger” mutations. These “driver” mutations have recently been classified by Vogelstein et al. [93] into “mut-driver” and “epi-driver” mutations, i.e. mutations to the primary DNA sequence and epigenetic modifications respectively. We propose that a third category of driver should be added to this classification: the exogenous drivers provided by oncogenic viruses, which could be called ‘vir-drivers’. This term denotes tumour drivers derived from viral sequences integrated in the host genome, such as the promoter-enhancer in a retroviral LTR or a gene product encoded by the viral nucleic acid (e.g. HTLV-1 Tax protein or HBZ mRNA, or HPV E6 and E7 proteins). The presence in cases of ATLL of genetic deletions and point mutations of the provirus that occurred before integration suggests that a solitary 3′ LTR of HTLV-1 is sufficient to act as a vir-driver.

In this review we have defined HTLV-1-infected T-cell clones on the basis of their proviral integration site, which indicates a common cell of origin. There is emerging evidence of the importance of subclones in cancer. Recent single-cell sequencing analysis of renal cell carcinomas and myeloproliferative disorders has identified minor subclones carrying rare genetic changes that may contribute to tumour progression. Aggressive ATLL is characterized by rapid relapse following chemotherapy; however, it remains unknown whether such relapse is more commonly due to the emergence of subclones of the same malignant clone that carry additional genetic changes (tumour drivers) or rather to clonal succession [77]. Whichever mechanism operates, it may be necessary to target more than one molecular pathway simultaneously, to reduce the chances that the disease becomes refractory to treatment, as in the treatment of solid tumours and persistent infections such as tuberculosis. Repeated and multiple site sampling for genetic analysis may become routine in monitoring the response to treatment in both solid and haematological malignancies.

## Conflict of interest

None declared.
